# Expression of Concern: MicroRNA-9 inhibits high glucose-induced proliferation, differentiation and collagen accumulation of cardiac fibroblasts by down-regulation of TGFBR2

**DOI:** 10.1042/BSR-2016-0346_EOC

**Published:** 2023-01-19

**Keywords:** cardiac fibrosis, high glucose, human cardiac fibroblasts, miRNA-9, TGFBR2

The Editorial Office has been made aware of potential issues surrounding the scientific validity of this paper, hence has issued an expression of concern to notify readers whilst the Editorial Office investigates. Some of the western blots seem to appear in other papers by unrelated authors. These include the a-tubulin blots from Figure 6A, which also appear as the Figure 2F a-tubulin blots from Chen et al. 2019 (doi: 10.3727/096504018X15251282086836); the Figure 5A TGFBR2 blots appear as the Figure 5A PIK3R3 bands from Sun et al. 2020 (doi: 10.1042/BSR20193867); and the Figure 2 blots appear in Figure 3B from Wang et al. 2018 (https://doi.org/10.1186/s11658-018-0089-x).

